# Characterisation of Polypropylene Composite Reinforced with Chemi-Thermomechanical Pulp from Oil Palm Trunk via Injection Moulding Process

**DOI:** 10.3390/polym15061338

**Published:** 2023-03-07

**Authors:** Chuan Li Lee, Kit Ling Chin, Paik San H’ng, Pui San Khoo, Mohd Sahfani Hafizuddin

**Affiliations:** 1 Institute of Tropical Forestry and Forest Product, Universiti Putra Malaysia, Serdang 43400, Malaysia; 2 Faculty of Forestry and Environment, Universiti Putra Malaysia, Serdang 43400, Malaysia; 3 Centre for Advanced Composite Materials, Universiti Teknologi Malaysia, Johor Bahru 81310, Malaysia

**Keywords:** wood plastic composite, oil palm trunk, chemi-thermomechanical pulp, polypropylene, injection moulding, material formulation, processing parameter

## Abstract

As the products made from wood–plastic composites (WPCs) become more sophisticated and required more detail profiles, the injection moulding processing method with wood pulp as the reinforcing material is the answer to cater to the rapid change and demands of composite products. The general objective of this study was to study the effects of the material formulation, as well as the injection moulding process parameters, on the properties of a polypropylene composite reinforced with chemi-thermomechanical pulp from oil palm trunks (PP/OPTP composite) via the injection moulding process. The PP/OPTP composite with a material formulation of 70% pulp/26% PP/4% Exxelor PO produced using injection moulding at 80 °C as the mould temperature and with 50 tonnes of injection pressure exhibited the highest physical and mechanical properties. The increment loading of pulp increased the water absorption capacity of the composite. Higher loading of the coupling agent effectively reduced the water absorption capacity and increased the flexural strength of the composite. The increase in mould temperature from unheated to 80 °C prevented excessive heat loss of the flowing material, which enabled the molten material to flow better and filled up all cavities in the mould. The increased injection pressure slightly improved the physical properties of the composite, but the effect on the mechanical properties was insignificant. For the future development of WPCs, further studies should be focused on the viscosity behaviour, as a greater understanding of the processing parameters’ effects on the PP/OPTP’s viscosity behaviour will lead to improved product design and enable great potential usage of WPCs.

## 1. Introduction

The raw materials used in wood plastic composites are mainly wood fibre–wood powders and thermoplastic polymers such as polyethylene (PE), polyvinyl chloride (PVC), polypropylene (PP), polystyrene (PS), and acrylonitrile–butadiene–styrene (ABS) [1,2]. Additionally, compatibilizers or coupling agents are added to improve the properties of WPCs. The performance of a WPC depends on the type and content of wood filler, the choice of polymer matrix, and the compatible technology and process parameters [3]. Since wood materials are hydrophilic and plastic substrates are hydrophobic, the mechanical properties of the filler are not fully utilised due to the poor bonding effect during production. Therefore, the main principle for improving the mechanical properties of a WPC is to enhance the interface bonding force between the wood materials and plastic substrates. The main methods used to enhance the interfacial bonding strength are surface pretreatments of the wood material, including physical and chemical methods [4,5,6].

Wood flour is the most used reinforcing biomaterial for the production of composites with thermoplastics. However, the interest in utilising thermomechanical pulp (TMP) instead of wood flour for the manufacture of wood–plastic composites (WPCs) has grown in recent years due to several advantages. TMP has a higher ratio of the length to diameter (aspect ratio), and this modified wood fibre also results in improved compoundability and decreased melt viscosity compared to wood flour [7,8]. TMP is typically produced for medium-density fibre boards (MDF) using a refining process. Improvements in strength properties were found using TMP with a higher aspect ratio compared to wood flour as a reinforcing material [9]. Compared to the use of wood flour as the filler, WPCs using TMP showed the best mechanical property retention after reprocessing due to the having lowest reduction in the TMP fibre length after six cycles [10]. So as to reduce the harsh effect on the fibre quality and to intensify efforts to increase energy efficiency during the TMP refining process, low-severity chemical pretreatments such as NaOH were applied to soften the wood before the refining process, with the aim of creating more individual wood fibres during the processing [11], with the produced pulp known as chemi-thermomechanical pulp (CTMP). Without pretreatment, harsh defibration conditions may lead to thermal degradation and shortening of the fibres. Lerche et al. [12] investigated the effects of defibration conditions on the mechanical and physical properties of WPCs and revealed that mild defibration conditions are advantageous, because of their tendency for lower thermal wood degradation and less lignin-covered fibre surfaces.

Wood fibres with a long or rather high aspect ratio have a reinforcing effect on composites. Migneault et al. [13,14] tested extrusion WPCs reinforced with CTMPs of different sizes and revealed that fibres with a high aspect ratio had a positive effect on the mechanical and physical properties. Nygard et al. [15] studied TMP- and CTMP-based extrusion WPCs and found that CTMP led to slightly higher tensile and impact strengths than TMP due to the fewer lignin-covered surfaces of the CTMP. During CTMP pulp production, massive changes occurred in the physical and chemical properties of the fibres, including the breakage of the hydrogen bonds in cellulose and removal of some of the lignin and wax covering the natural fibres; these changes may be beneficial for obtaining composites with improved characteristics. Changes in the fibres’ chemical composition will affect the fibre matrix adhesion or the mode of action of certain coupling agents [7,16]. Free radical formation in various modifications of wood cellulose accompanied by the chemical method of graft copolymerisation is one of the possible routes to increase the potential usage of WPCs. Chemical grafting using maleic anhydride (MA) is the most popular treatment not only to modify fibre surfaces but also the thermoplastic matrix to achieve better interfacial bonding and mechanical properties in composites [17]. It exploits the hydroxyl groups that are abundantly available in the wood fibres from one side and grafts to the matrix polymer from the second side [18]. Correspondingly, grafted MA, like maleic-anhydride-grafted polypropylene (MAPP), can be utilised directly with the available compounding machine without inserting a new processing step into the production cycle [17].

The extrusion method is the most common method used to produce WPC products [19]. However, extruded WPC products are restricted to profiles with a uniform cross-section. The most common extruded WPC products are decking, railing, and flooring products. Due to the limitation of the extrusion process, the market shows a trend of developing WPC products using the injection moulding process. Injection moulding process basically involved pressurising the molten materials into a cavity mould to form a different shaped products. This process is mostly found in the automotive industry. The advantages of applying the injection moulding process in WPC production are the vast shape-making capability, high productivity, production of precise components with complex shapes, and low processing cost [20]. Hence, injection moulding is a great alternative for WPC processing technology, where flexibility in the design of the product can be considered as value added to the WPC products.

The physical and mechanical properties play important roles in determining the WPC’s applications and usages. These properties are the guidelines for the industry for its use as an alternative and cheaper solution replacement or for potential industrial applications [21]. The specific material formulation of the products is the key factor in their success. Decking, siding, and fencing products are some of the successful WPC products that have gained market acceptance, as well as other potential advanced WPC products such as pallets, shims, grading stakes, and office accessories [22], where the precise processing parameters are controlled to produce composites with specific applications. WPCs from the extrusion process have been extensively studied. However, with the recent development of the injection moulding process for products made of thermoplastic materials with cross-profiling, there is a need to understand the materials’ formulation and process parameters for this specific WPC production method.

The CTMP from oil palm trunks (OPTs) used as the reinforcing material in this study is a lignocellulosic-rich material that has numerous qualities, such as being inexpensive and renewable. Despite being utilised in various ways, such as in laminated panels, OPT products have yet to advance into the competitive market of bioproducts. However, the utilisation of OPT for injection-moulded WPC products could be an alternative method to reduce the environmental impact of OPT waste and to remain viable in the competitive market of biocomposites. As the product design process for WPCs becomes more sophisticated and requires a detail profile, the injection moulding processing method is the answer to cater to the rapid changes and demands of WPC products. Injection moulding is a comprehensive solution for WPC development due to the unrestricted shape moulding possibilities, better compaction, and better fibre/matrix adhesion compared to extrusion moulding. However, there are two important aspects that should be analysed as the fundamental approach to producing WPCs using an injection moulding processing system. These aspects include (i) a suitable material formulation for the WPC to achieve the required physical and mechanical properties to suit the general applications and (ii) optimum injection moulding process parameters to produce a WPC with high physical and mechanical properties. The general objective of this study was to study the effects of the pulp loading process, pulp type, and pulp loading percentage of the coupling agent on the properties of a polypropylene composite reinforced with CTMP from oil palm trunks (PP/OPTP composite) via injection moulding, as well as to analyse the optimum injection moulding process parameters (the injection pressure and mould temperature setting) to maximise the physical and mechanical properties of the WPC composite.

## 2. Materials and Methods

The study of a polypropylene composite reinforced with chemi-thermomechanical pulp (PP/OPTP composite) produced using the injection moulding process was separated into two parts: (i) the effect of the material formulations on the physical and mechanical properties of the PP/OPTP composite; (ii) the effect of the injection moulding parameter settings on the physical and mechanical properties of the PP/OPTP composite.

### 2.1. Preparation of OPT Pulp (OPTP)

**Figure 1:**
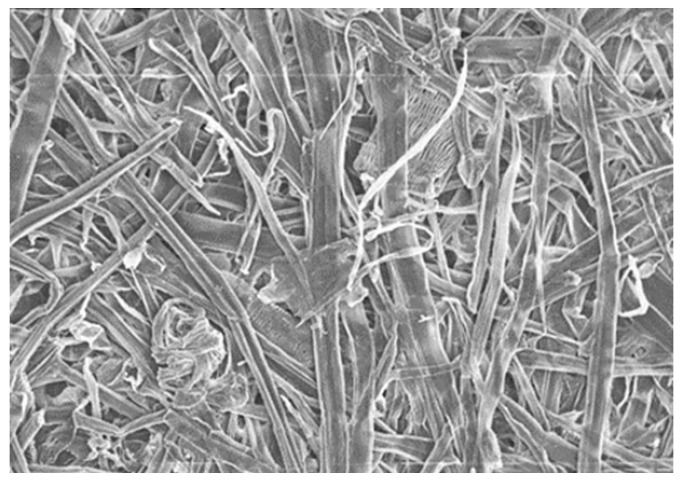
Scanning electron micrographs of OPT chemi-themomechanical pulps at 200× magnification.

Thirty-year-old unproductive oil palm trees (*Elaeis guineensis*) were obtained from an oil palm plantation in Universiti Putra Malaysia, Serdang. The OPTs were chipped and screened to a chip size of approximately 1.5–2 cm^3^. The OPT chips were immersed in a sodium hydroxide (NaOH) (Fisher Scientific Co LLC, Loughborough, United Kingdom) solution at a concentration of 3% for 24 h before the thermal pretreatment. The thermal pretreatment of the OPTs was carried out by steaming the chips at 170 °C for 10 min. These pretreated fibres were then passed through a refiner (Andritz Sprout Bauer Inc., Muncy, PA, United States) twice with a disk gap of 2.5 mm (two refining stages). The refined pulps were then screened using a Somerville Screener (Paper Testing Instruments Ltd., Laakirchen, Austria) with a 0.25 mm mesh screen. With this, a material with more homogeneous particles was obtained. After the refining process, the OPT pulps were spin-dried to remove excessive water and oven-dried at 80 °C for 24 h. The properties of the OPT pulp used in this study are summarised in Table 1 and the SEM image was shown in Figure 1.

### 2.2. Fabrication of Polymer Composite Reinforced with OPT Pulp (PP/OPTP Composite)

The main properties of the polypropylene (Homopolymer G452) used in this study are shown in Table 2 and were obtained from Propelinas, Malaysia. Three types of maleic-anhydride-based coupling agents (Exxelor PO 1020 (EX), CA 10512 (CA), and Epolene E-43 (EP)) were used in this study. The Exxelor PO 1020 was supplied by ExxonMobil Chemicals, Kuala Lumpur, with a melt flow rate of 119 g/10 min (190 °C/1.2 kg). The CA 10512 was supplied by TLK Polytech, Selangor, with a melt flow index rate of 10 g/10 min (190 °C/0.325 kg), and the Epolene E-43 was supplied by Lotte Chemical Titan, Selangor.

### 2.3. Formulating the Material Loading Percentage for the Injection Moulding Process

Table 3 shows the variables used in determining the material loading formulations to produce a polymer composite reinforced with OPT pulp (PP/OPTP composite) with high flexural properties and low water absorbance properties. The variables selected for this study were the loading percentage of the OPT pulp, loading percentage of the polypropylene (PP), and loading percentage of the different coupling agents. Three replicate boards were produced for each composite formulation.

*Premixing*: In the plastic and additive premixture preparation process, different formulations of plastic and additive were prepared separately. The polypropylene (PP) and coupling agent were loaded into the plastic mixer based on the ratios shown in Table 3. The mixing plastic was premixed for 30 to 60 min. Next, the premixtures were packed and labelled. The pellets were produced in the compounding process using a compounding machine with a co-extruder combination. The plastic and additive premixture was poured into the co-extruder, which was designed to premelt the plastic and the plastic base additives before mixing with the pulp. This was to ensure the plastic was fully melted before mixing to obtain a constant and uniform mixture.

*Compounding process*: The main extruder section was built-in with a twin screw and barrel with a heating control element. In this section, the OPT pulp was fed and traversed under pressure at 180 °C. The pulp was further dried and heated up to a temperature of around 180 °C before mixing with the melted plastic. The mixing process in this compounding section produced a mixture of materials prior to the pelletising process.

*Pelletising process*: The mixture of materials was later delivered to the pelletiser machine to form the pellets. During the pelletising process, the mixture of materials was pressed and flowed through a Ø6.0 mm hole to form pellets with a Ø6.0 mm cylinder size. The pellet length can be predetermined by adjusting the cutter at the outlet of the pelletiser machine. In this study, the pellets length range was set to 10–15 mm.

*Injection moulding process*: The pellets were loaded into twin-screw extruder hopper and melted at the temperature of 180 °C. This was a very important process to ensure the pellets were fully melted to the required temperature before being transferred for the injection process. The extruder barrel temperature was set to 180 °C with an extrusion speed of 5 rpm. The melted material was collected manually and weighed prior to the injection moulding process. The press cylinder barrel was set to 180 °C and preheated for 10 min before the material was loaded. The press cylinder was mounted on top of the injection mould and the piston was mounted together with the top press plate of the hydraulic press machine. The melted material with the required weight was manually loaded into the press cylinder barrel. The melted material was injected into the mould and pressed at the pressure of 40 tonnes using a hydraulic press machine. After the pressing time of 10 s, the material was left to cool and solidify inside the mould for 4 to 5 min. After cooling, the composite was removed from the mould.

*Board conditioning*: The board was conditioned at 20 ± 2 °C and 65 ± 5% relative humidity for 1 week in accordance with ASTM Standard D1037-99. The conditioning process was carried out for all the test samples to obtain a uniform moisture content in the board.

### 2.4. Setting of the Injection Moulding Process Parameters

This part of the study focused on the setting of the injection moulding process parameters by analysing the effects of the injection moulding parameters and material loading formulations on the physical and mechanical properties of the composite. Only a selected type of coupling agent and loading ratio were used in this optimisation study. The selected coupling agent was based on the results obtained from the first part of the study (Section 3.1), where the formulation of materials produced composites with the best physical and mechanical properties. The effects of the injection moulding process parameters, including the mould temperature (25 °C, 50 °C, and 80 °C), pressing pressure (40 and 50 tonnes), and the pulp loading percentage (65, 70, and 75%) on the physical and mechanical properties of the composite were determined. Three replicate boards were produced for each composite formulation. The control parameters of the injection moulding process for this study are shown in Table 4.

### 2.5. Evaluation of the Polymer Composite Reinforced with OPT Pulp

The PP/OPT boards were cut into specimens to be used in the physical and mechanical tests. Three replicates of PP/OPT boards were produced for each composite formulation or treatment. The total numbers of specimens for each sub-study are listed below.

For the study on the effect of the material formulations, there were 27 formulations. Three replicate boards were produced for each composite formulation, so the number of boards produced was 81. From each board, five replicate specimens were prepared for each evaluation test (density and moisture content, water absorption, static bending). The total number of replications for each formulation was 15, totalling 405 specimens for all treatments.

For the study on the effect of parameter settings on the injection moulding process there were 18 treatments. Three replicate boards were produced for each treatment, so the number of boards was 54. From each board, five replicate specimens were prepared for each evaluation test (density and moisture content, water absorption, static bending). The total number of replications for each formulation was 15, totalling 270 specimens for all treatments.

#### 2.5.1. Density

The density of the PP/OPTP specimens measuring 50 mm in length by 50 mm in width with a board thickness of ≈3 mm was tested according to ASTM D 2395-14 [23]. The specimens were air-dried in the conditioning room at 20 ± 2 °C and 65 ± 5% relative humidity. A weighing balance was used to weigh the mass of each PP/OPTP specimen. The volume of the specimens was measured by multiplying the specimen’s length, width, and thickness. The below equation was used to calculate the density of the specimens:

Density (g/cm^3^) = (Mass)/Volume (1)

where Mass is the mass of the specimen in g and Volume is the volume of the specimen in cm^3^.

#### 2.5.2. Moisture Content

The moisture content of the specimens measuring 50 mm in length by 50 mm in width with a board thickness of ≈3 mm was tested according to ASTM D 4442-07 [24]. The specimen’s initial weight was recorded. The specimens were then oven-dried at 103 ± 2 °C until a constant weight was achieved and weighed at an accuracy of ± 0.01 g. Equation (2) was used to calculate the moisture content of the specimens.

Moisture content (%) = (W_I_–W_O_)/W_O_ × 100 (2)

where W_I_ is the initial weight of the sample and W_O_ is the oven-dried weight of the sample.

#### 2.5.3. Water Absorption

Specimens measuring 50 mm in length by 50 mm in width with a board thickness of ≈3 mm were weighed before being submerged horizontally in 25 mm of distilled water at a temperature of 20 ± 1 °C. The water was removed after 22 h of submersion. The specimens were suspended to drain for 10 ± 2 min to remove excess surface water and were then weighed. The specimens were then placed in an oven at 103 ± 2 °C to determine the moisture content based on oven-dried weight. According to ASTM D 1037-12 [25], the absorbed water in the specimens was calculated as a percentage. Equation (3) was used to calculate the percentage of water absorption:

Water absorption (%) = (W_I_–W_O_)/W_O_ × 100 (3)

where W_I_ is the initial weight of the sample and W_O_ is the oven-dried weight of the sample.

#### 2.5.4. Flexural Test

Flatwise three-point static bending tests were performed on specimens measuring 122 mm in length by 50 mm in width with a board thickness of ≈3 mm using a universal testing machine (Bluehill Instron 5567; Instron, Shakopee, MN, USA) in accordance with ASTM D4761 [26]. Throughout the test, the crosshead loading speed was maintained at 1.05 mm/min. Equations (4) and (5) were used to calculate the modulus of rupture (MOR) and modulus of elasticity (MOE) for each specimen.

(4) $$\mathrm{MOR},\;\frac{N}{{\mathrm{mm}}^{2}}=\frac{3\mathrm{PL}}{2{\mathrm{bd}}^{2}}$$

(5) $$\mathrm{MOE},\;\frac{N}{{\mathrm{mm}}^{2}}=\frac{P_{1}L^{3}}{4{\mathrm{bd}}^{3}Y_{1}}$$

where:

P = maximum load, in Newtons;
P_1_ = load at the proportional limit, in Newtons;
Y_1_ = center deflection at the proportional limit load, in mm;
L = length of the span, in mm;
b = width of the specimen, in mm;
d = thickness of the specimen, in mm.

### 2.6. Statistical Analyses

The statistical analyses were carried out using the statistical package SPSS for Windows, version 16.0 (SPSS, Chicago, IL, USA). An analysis of variance (ANOVA) was used to evaluate the effects of the material formulations and processing parameters on the physical and mechanical properties of the PP/OPTP. A Tukey–Kramer multiple comparisons test was applied to analyse the differences between the treatment effects when significance was observed. The effects were considered to be not statistically significant when the *p*-value was higher than 0.05 at the 95% confidence level. This study was separated into two smaller sections: (i) the effects of the material formulations on the physical and mechanical properties of the composites produced using the injection moulding process; (ii) the effects of the injection moulding parameters on the physical and mechanical properties of the composites produced using the injection moulding process. The dependent and independent variables selected in the two study sections were as listed below.

(i) To study the effect of material formulations on the physical and mechanical properties of PP/OPTP composite, the pulp loading percentage (65, 70, and 75%), type of coupling agent (Exxelor PO 1020, CA 10512, and Epolene E-43), and coupling agent loading percentage (2, 3, and 4%) were chosen as the independent variables. The water absorption, MOR, and MOE values of the produced boards were used as the dependent output variables. The study was conducted as a 3 × 3 × 3 factorial experiment with a completely randomised design (CRD) and subjected to an ANOVA.
(ii) To study the effects of the injection moulding parameters on the physical and mechanical properties of the PP/OPTP composite, the pulp loading percentage (65, 70, and 75%), temperature (25 °C, 50 °C, and 80 °C), and pressing pressure (40 and 50 tonnes) were chosen as the independent variables. The water absorption, MOR, and MOE values of the produced boards were used as the dependent output variables. The study was conducted as a 3 × 3 × 2 factorial experiment using a completely randomised design (CRD) and subjected to an ANOVA.

## 3. Results and Discussion

The results of this study were separated into two sections: (i) the effects of material formulations on the physical and mechanical properties of the composites produced using the injection moulding process; (ii) the effects of the injection moulding parameters on the physical and mechanical properties of the composites produced using the injection moulding process. The coupling agent and the loading percentage that produced the optimum physical and mechanical properties (results presented in Section 3.1) were further studied to analyse the optimum injection moulding condition, the results of which are presented and discussed in Section 3.2.

### 3.1. Effects of Material Formulations on the Physical and Mechanical Properties of the PP/OPTP Composite

#### 3.1.1. Moisture Content and Density

The mean values in terms of the moisture content and density of the PP/OPTP composites with different material formulations are summarised in Table 5. The moisture content rates of the composite test samples ranged from 0.47% to 1.02%, while the density values of the composite test samples ranged from 1.11 to 1.22 g/cm^3^.

From the ANOVA results, no significant differences (*p* > 0.05) were observed among the variables (pulp loading × type of coupling agent × coupling agent loading) in terms of the boards’ moisture content and density values. Furthermore, significant differences (*p* ≤ 0.05) were only observed in the moisture contents for different levels of pulp loading. This indicated that the pulp loading affects the moisture content independently. The moisture contents of the produced composites increased slightly when the pulp loading percentage increased. This may be attributed to the high absorption capacity of the pulp fibre lumen [27]. The board density was not significantly affected by the usage of different levels of pulp loading, the type of coupling agent, or the loading percentage (*p* > 0.05). The board density mainly contributed to the compaction of the raw materials during injection. As the pressing pressure remained constant in this study, there were not much of a compaction change with the different material formulations (treatments); hence, no significant changes were observed in terms of density.

#### 3.1.2. Water Absorption Analysis

The water absorption of the composites was measured and the values obtained ranged from 0.85% to 2.96% (Table 6). From the ANOVA results, highly significant differences (*p* ≤ 0.01) were observed for all variables (pulp loading, type, and loading percentage of the coupling agent) in terms of the water absorption of the composite boards. Significant interactions (*p* ≤ 0.01) among the variables (pulp loading × type of coupling agent × coupling agent loading) in terms of the water absorption of the composites were also observed and are shown in Table 6. Here, the 4% coupling agent concentration provided the composite material with better water resistance compared to the lower coupling agent concentration. However, the composite produced with a 75% pulp loading resulted in higher water absorption (2.53%) compared to the composite produced with 65% pulp loading (1.37%). The water absorption capacity was significantly increased with the increased pulp loading. As the pressing pressure remained constant in this study, there was not much of a compaction change with the increase in pulp loading. This could be observed from the non-significant differences in results in terms of the density with higher pulp loading. The higher water absorption capacity of the PP/OPTP composite with the high pulp content may be attributed to the amount of water absorbed by the cellulose fibres in the pulp, which is influenced by the void volume and the amount of cellulose material that is present. The hydrophilic nature of the natural fibres (free hydroxyl groups) caused an increase in the water absorption. These hydroxyl groups interact strongly with water molecules via hydrogen bonding, which favours water absorption [28]. As the pulp loading percentage in the composite board decreased from 75% to 65%, the water absorption capacity was significantly reduced. This may have been due to the decreased volume of the pulp loading causing more pulp to be encapsulated by the plastic, thereby reducing the exposure of the pulp to moisture [6].

The Exxelor PO 1020 produced PP/OPTP boards with a significantly lower water absorption capacity compared to the other two coupling agents (CA 10512 and Epolene E-43) used in this study. Overall, the water absorption capacity was reduced when the loading percentage of the coupling agent increased, regardless of the type of coupling agent. The increased coupling agent loading percentage improved the molecule bonding in the composite, which reduced the structural cracking due to the hygrothermal expansion of the fibres in the composite board against time [29]. The coupling agent helps to stabilise the PP/OPTP bonding against the hygrothermal effect. Therefore, the increased coupling agent loading percentage would help in minimising the cracking of the PP/OPTP, as well as minimising the exposure of pulp fibre surfaces to water. Therefore, the water absorption of the composite board can be reduced when there are fewer surface cracks with a higher coupling agent loading percentage.

#### 3.1.3. Mechanical Properties

The analysis of variance revealed highly significant differences (*p* < 0.01) in MOR and MOE values for the PP/OPTP composite based on the effects of the different levels of pulp fibre loading, the types of coupling agents, and the loading percentages. Significant interactions at *p* ≤ 0.01 for all independent variables (pulp loading × type of coupling agent × coupling agent loading) on the MOR and MOE are depicted in Table 6. The Tukey–Kramer multiple comparison test was employed to determine the interactions among the independent variables.

The highest MOR (63.3 MPa) values were for the 70/26/4EX panel type (4% of Exxelor PO 1020 with 70% pulp loading), followed by the 75/21/4EX (4% of Exxelor PO 1020 with 75% pulp loading) and 65/31/4CA panel types (4% of CA 10512 with 65% pulp loading). The lowest MORs were observed for the panel types 70/28/2EX (2% of Exxelor PO 1020 with 70% pulp fibre loading), 75/23/2EP (2% of Epolene E-43 with 75% pulp loading), and 75/22/3EP (3% of Epolene E-43 with 75% pulp loading). Panel types 70/26/4EX (4% of Exxelor PO 1020 with 70% pulp fibre loading) and 75/21/4EP (4% of Epolene E-43 with 75% pulp loading) gave the highest MOE values of 4275 MPa and 4270 MPa, respectively. Panel type 65/33/2EX (2% of Exxelor PO 1020 with 65% pulp loading) gave the lowest MOE value.

In terms of the flexural properties, panel type 70/26/4EX (4% of Exxelor PO 1020 with 70% pulp fibre loading) gave the highest MOR (4275 Mpa) and MOE (63.3 MPa) values as compared to the other panel types. Exxelor PO1020 was more efficient than CA10512 and Epolene E-43 as coupling agent for the PP/OPTP composite in this study.

The use of Exxelor PO1020 in the PP/OPTP composite significantly increased the MOR as compared to the other two coupling agents. Both CA1051 and Epolene E-43 obtained better MOR performance results at 3% and 4% concentrations with 65% WF loading. The coupling agents CA 10512 and Epolene E-43 were unable to accommodate higher pulp contents, as the composite boards produced with pulp loadings higher than 65% resulted in lower MOR values than composites made with 70% and 75% pulp loadings. Even increasing the loading percentages of these two coupling agents (CA 10512 and Epolene E-43) did not greatly increase the MOR.

Mazzanti et al. [30] revealed that the optimum fibre loading is required during the reinforcement of fibre with plastics. Beyond the optimum point, it is difficult for the fibre–plastic mixture to flow and mould properly, especially when the type and loading percentage of the coupling agent do not positively contribute. The difficulty of the material flow will result in inconsistency in the PP matrix distribution during the sample formation. This will cause poor interfacial bonding between the pulp fibre and PP matrix and inefficient stress transfer when strain is exerted on a specimen. This may explain why the WPC produced with 70% pulp loading performed significantly better than the 65% and 75% pulp loadings. Generally, from the statistical analysis, composites compounded with 4% coupling agent produced better flexural performance results. By loading 4% of the coupling agent, the MOR increased, and this may have been due to the improvement that occurred between the interaction of the pulp fibre and PP in the composite. Anbupalani et al. [29] stated that a higher coupling agent loading percentage effectively improved the bonding between the pulp fibre and PP molecules by reacting as a coupling agent between both the anhydride and hydroxyl matrix, which completely strengthened the bonding.

Most of the extracts were removed during the CTMP process. Following the removal of extracts, this was attributable to an improvement in flexural properties, with an improvement in interfacial bonding being suggested as the mechanism [31]. The improvement in the flexural properties of PP/OPTP composites was likely due to a combination of (a) improved mechanical interlocking due to the fibre wall swelling and roughness and (b) improved interfacial adhesion due to extract removal [32,33,34].

Based on the result, this shows that the MOE increased with the increasing pulp loading. As stated by Dolza et al. [20] and Ashori et al. [35], the increase in MOE was the most prominent physical effect caused by fibre loading in WPCs. This is due to the modulus of the fibres being more rigid than for plastic; therefore, the energy required to deform the composite during the bending test increases as the fibre loading increases. The loading percentages of 3% and 4% coupling agent performed better than 2% coupling agent in terms of the MOE. The results showed that the highest MOE values were obtained from composites produced with 4% coupling agent (Exxelor PO 1020) and 70% or 75% pulp loading.

**Figure 2:**
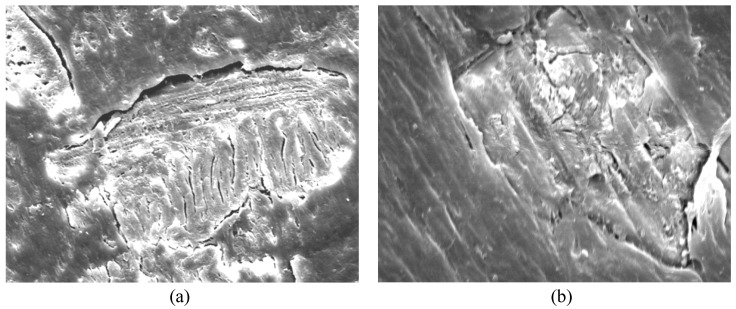
Scanning electron micrographs of PP/OPTP composites from 70% pulp loading with (**a**) 2% Exxelor PO 1020 and (**b**) 4% Exxelor PO 1020 at 2000× magnification.

The composite with a lower loading percentage of coupling agent (70% pulp/28% PP/2% Exxelor PO 1020) exhibited bigger voids and cracks in comparison to the composites with higher loading percentages of coupling agent (Figure 2a). The composite formulated with a higher coupling agent percentage (70% pulp/28% PP/4% Exxelor PO 1020) showed better encapsulation of the PP matrix over the pulps, whereby smoother surfaces and smaller cracks were observed (Figure 2b). Pulp particles were less obvious on top of the surfaces of composites with higher loading percentages of coupling agent. When adding a higher percentage of coupling agent, the number and dimensions of cracks within the structure were reduced. The addition of coupling agents increased the wettability between the pulp and the PP, enhanced the dispersion in the matrix, and improved the adhesion in both components [29]. With the dispersion enhancement, this promotes better encapsulation of the pulp fibres and the polymer matrix, reducing the water absorption in the composites [36]. Moreover, the improvement of the mechanical properties of the composite with higher loading of the coupling agent could be an explanation.

### 3.2. Effects of Injection Moulding Parameter Settings on the Physical and Mechanical Properties of PP/OPTP Composite

From the results presented in Section 3.1, it is shown that higher physical and flexural properties were obtained by adding 4% of Exxelor PO 1020 into the material formulation. This type and loading percentage of coupling agent were applied in the production of the PP/OPTP composite discussed in this section to further study the effects of the injection moulding parameters (mould temperature and injection pressure) on the physical and mechanical properties of the composite.

The moisture content of the PP/OPTP composites produced from different treatments was in the range of 0.66–0.91%. From the ANOVA result, significant differences (*p* ≤ 0.05) were only found in the moisture content for the different levels of pulp loading and different mould temperatures used in the injection moulding process for the PP/OPTP composite boards. The different levels of pressure showed no significant effects (*p* > 0.05) on the moisture content. No correlation (*p* > 0.05) was observed among the variables (pulp loading x mould temperature x injection pressure) based on the moisture content (Table 7). Increasing the mould temperature during the injection process slightly reduced the final moisture content of the produced composites. As the mould temperature used in the injection moulding process increased from room temperature to 80 °C, the final moisture contents of the composites reduced from 0.72 to 0.66% for the 65% pulp loading, from 0.86 to 0.72% for the 70% pulp loading, and from 0.92 to 0.76% for the 75% pulp loading. As the pulp fibre and plastic mixture distributed in the mould, the heated mould could have acted as a dryer and may have reduced the moisture content of the pulp fibres, which overall may have reduced the moisture content of the composite.

The density of the PP/OPTP composites produced from different treatments was in the range of 1.11–1.22%. A significant difference (*p* ≤ 0.05) was only revealed in the board density values for different levels of mould temperature and different pressures. No correlation (*p* > 0.05) was revealed among the injection moulding variables (pulp loading × mould temperature × injection pressure) based on the density (Table 7). This indicated that the mould temperature and pressing pressure affect the density independently. The density increased as the mould temperature and injection pressure increased. This showed that the higher mould temperature of 80 °C created higher compaction of material the loading; hence, a higher density composite was produced compared to the mould temperatures of 50 °C and room temperature. The increase in density with the increased of mould temperature was mainly affected by the viscosity of the material during the injection process. With the higher mould temperature, the molten material injected into the mould retained its low viscosity and easily flowed to cover the cavities in the mould during the injection process [37]. Therefore, the PP/OPTP mixture was more evenly distributed when compressed in a mould set at a higher temperature compared to a lower temperature mould.

The composite density also showed improvements with the increase in pressing pressure. The increased pressing pressure increased the material packing pressure during the material solidification process [38]. During injection moulding, pressure not only acts as the main force to feed the molten material into the cavity mould, it also provides the packing force to compress the liquid polymer to penetrate the hollow fibres, known as the bulk packing effect. This force is also used as the holding force to hold the mould and secure the material inside the cavity mould during the material solidification process. Therefore, it provides more compression force to the molten material and creates more molten material to be compressed during formation. This would increase the molecular weight of the PP/OPTP during injection. As a result, the density of the composite would be increased as well.

#### 3.2.1. Water Absorption

The water absorption levels of PP/OPT composites samples were between 0.90% and 2.25%. From the analysis of variance, highly significant differences (*p* < 0.01) were observed for the effect of each injection moulding parameters (pulp loading, mould temperature, and injection pressure) based on the water absorption capacity of the PP/OPTP composites. A significant interaction (*p* < 0.01) amongst the variables studied (pulp loading × mould temperature × injection pressure) was also found.

As shown in Table 8, the pulp loading and mould temperature had a more dominant effect than the injection pressure on the water absorption capacity of the PP/OPTP composites. The composite with 60% pulp loading with an 80 °C mould temperature showed lower water absorption (0.90%), whereas the 75% pulp loading with an unheated mould had a higher water absorption capacity of 2.25%. As mentioned earlier, a higher pulp loading will create a higher surface area containing pulp that attracts water; therefore, the water absorption will increase as the pulp loading increases.

Additionally, as the mould temperature increased, the water absorption was reduced. The same effect was also observed for the higher injection pressures we applied. Higher mould temperatures and injection pressures reduced the water absorption capacity of the PP/OPTP composites. This may have been due to the better compaction of the loaded materials, as both variables allowed the pulp–plastic mixture to flow easier and fill the mould cavity with a better distribution. Lower injection pressures caused the polymer to flow more slowly during the formation, and as a result the polymer surface coverage was not consistent under lower injection pressures. Montanes et al. [38] stated that as the viscosity of the pulp–plastic mixture drops during the moulding process, this will create better flow of the melted materials and will improve the physical characteristics of the composite.

#### 3.2.2. Mechanical Properties

Table 8 shows the flexural properties of the WPC boards produced in this study under different processing parameters. The analysis of variance revealed highly significant differences (*p* < 0.01) in the flexural properties for the different levels of pulp loading and different mould temperatures used in the injection moulding process for the PP/OPTP composite boards, except for the injection pressures, which showed no significant differences (*p* > 0.05). Furthermore, no correlation among the injection moulding parameters (pulp loading x mould temperature x injection temperature) based on flexural properties was observed at the 95% confidence level. This indicated that the injection moulding parameters (pulp loading and mould temperature) affect the MOR and MOE independently. The mean values of the MOR and MOE for different levels of pulp loading and different mould temperatures were compared using the least significance difference (LSD) test at the 95% confidence level.

Table 9 shows the least significant difference (LSD) test results for the effects of the pulp loading on the flexural properties of the PP/OPTP composite. The composite with 70% pulp loading performed significantly better in terms of the flexural properties than the composites with 65 and 75% pulp loadings. As shown in Table 7, there were increases in the MOR and MOE values with the increases in mould temperature and injection pressure. The MOR values of the composites increased from 26.2 MPa to 28.2 MPa (7.6% increment) for the 65% pulp loading, from 63.3 MPa to 67.6 MPa (6.8% increment) for the 70% pulp loading, and from 42.2 MPa to 49.6 MPa (17% increment) for the 75% pulp loading. The MOE values increased from 2560 MPa to 2913 MPa (13.7% increment) for the 65% pulp loading, from 4238 MPa to 4898 MPa (15.6 increment) for the 70% pulp loading, and from 4240 MPa to 4725 MPa (11.45 increment) for the 75% pulp loading.

The result clearly show that the flexural performance of the PP/OPTP composites is strongly caused by the effect of the pulp loading in the composite. It is known that the flexural properties increase only up to a certain fibre content, with the 70% pulp loading being the cut-off point in this study. The flexural performance of a composite is the degree of fibre–plastic linkage, which refers to the bonding between the anhydride and hydroxyl matrix [39]. The linkage between pulp fibres and PP matrix can be enhanced by controlling the flow of the material and preventing the breakage of the pulp fibre–PP distribution during injection. The level of PP/OPTP viscosity depends on the pulp loading; the higher the pulp loading, the higher viscosity of the WPC material. This is due to less molten media being present to carry the pulp during the injection process. The increase in mould temperature becomes important as it able to reduce the viscosity of pulp fibre–plastic mixture and allow the material to flow easier on the molten surface [37].

Table 10 shows the significance level of the effect of the mould temperature on the flexural properties of the PP/OPTP composite. The composites produced at higher mould temperatures performed significantly better than those produced at lower mould temperatures during the injection moulding process. As mentioned earlier, higher temperature was required in the mould during the injection process, as it allowed the pulp–plastic mixture to flow smoothly into the mould and form the required shape. As the pulp–plastic matrix travels further from the injection nozzle, the mixture will become cooler, reducing the flow capability and compaction of the material. As the mould temperature increases to 80 °C, the heat provided from the mould will allow the pulp–plastic mixture to flow more easily and will reduce the shearing between the material and the mould. Increasing the mould temperature above the material–glass transition temperature leads to a reduction in the surface tension between the material in the mould and the material in the injection nozzle, which creates better adhesion. During injection moulding, molten PP/OPTP was forced to fill the mould cavity to form a composite board. The sudden drop in temperature from the injection nozzle to the mould caused the increase in material viscosity and also increased the material friction against the mould surfaces. The higher viscosity of the PP/OPTP material reduces the flowability against force, resulting poor flowability of the material in the mould. Due to this reason, the compaction of the material and degree of PP penetration into fibre surface will be significantly reduced with the increase in material viscosity caused by the increased temperature difference between the injection nozzle and the mould. Hence, controlling the temperature difference by adjusting the mould temperature affects the density as well as the physical and mechanical properties of the composite board.

**Figure 3:**
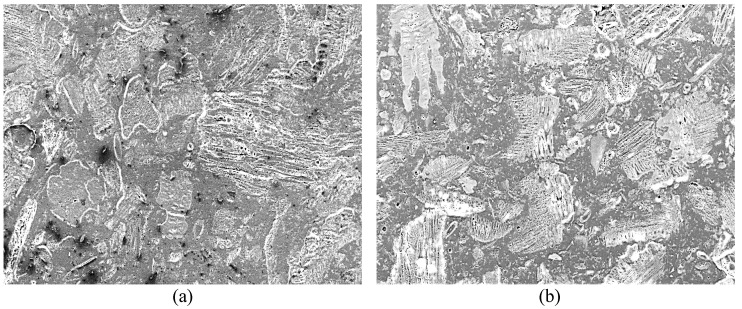
Scanning electron micrographs of the surfaces of PP/OPTP composites (70% pulp/26% PP/4% Exxelor PO 1020) produced with a pressing pressure of 50 tonnes and with (**a**) unheated and (**b**) 80 °C mould temperatures at 100× magnification.

As shown in Figure 3a, the poor distribution of the PP and pulp fibres with multiple voids on the surface was observed in the PP/OPT composite produced with an unheated mould during injection moulding. However, by increasing the mould temperature to 80 °C, the injection moulding process produced a PP/OPTP composite with less voids, a more homogeneous surface, and better encapsulation of the PP in the pulp fibre (as shown in Figure 3b). 

The injection-moulded PP/OPT composite produced using the material formulation of 70% pulp loading, 26% of PP, and 4% of Exxelor PO 1020, with a mould temperature of 80 °C and pressing pressure of 50 tonnes, was shown to obtain the highest physical and mechanical properties of 0.94% for the water absorption, 67.6 MPa for the MOR, and 4898 MPa for the MOE. Numerous studies using pulp as the reinforcing material for WPCs have been published and are depicted in Table 11. In general, it is difficult to compare the physical and mechanical results of these published studies with the current study due to the different production processes and material compositions. However, it could be observed that the pulp loading percentage has a significant influence on the physical and mechanical properties of the WPCs. WPCs highly filled with wood fibre and pulp have obvious advantages in terms of their environmental friendliness, application scenarios, and market competitiveness, and they are continuously being developed [40,41]. Most published papers have reported 60% as the maximum loading percentage of filler to produce WPCs with good properties [7]. However, in fact, the critical wood filler contents in WPCs usually depends on the processing technique. In order to improve the properties of WPCs with high filler contents, several modifications are needed, such as (i) physical modifications of the filler (e.g., surface pretreatment and increase aspect ratio), (ii) chemical modifications involving the addition of a coupling agent (for enhanced dispersion and interfacial adhesion between the filler and the polymer matrix), and (iii) modifications od the compounding and moulding process (for homogeneous fibre dispersion and minimal fibre breakage). Commercialised WPCs with specified properties are mostly used as deck boards and guardrail systems. As shown in Table 11, the properties of the produced WPCs in this study were comparable with the properties of the commercial WPCs that are used for floor decking.

## 4. Conclusions

The polypropylene composite reinforced with thermo-mechanical pulp (PP/OPTP composite) was evaluated as a potential material to reduce the use of plastic compounds. This study was performed to investigate the effects of the pulp loading, pulp type, and loading percentage of the coupling agent on the physical and mechanical properties of the PP/OPTP composite. On top of this, the enhancements of the injection moulding process parameters, such as the mould temperature control and injection pressure, were also evaluated. The PP/OPTP composite with a material formulation of 70% pulp/26% PP/4% Exxelor PO produced using an injection moulding temperature of 80 °C and 50 tonnes of injection pressure exhibited the best physical and mechanical properties. The increment in pulp loading percentage increased the water absorption capacity of the composite. At the level of 65% pulp loading, the composite was more resistant to water absorption compared to the 70% and 75% pulp loadings. However, the composite with 70% pulp loading performed better in terms of the MOR and MOE values than the composites with 65% and 75% pulp loadings. Higher percentages of coupling agent effectively reduced the water absorption capacity and increased the flexural strength of the composite. The increase in mould temperature from unheated to 80 °C during the injection process prevented excessive heat loss of the flowing material when filling the mould. With a heated mould, the molten material was able to flow better and filled up all cavities while maintaining the distribution of the PP and pulp fibre matrix. The increased injection pressure slightly improved the physical properties of the composite but the effect on the mechanical properties was insignificant. The further studies of PP/OPTP viscosity behaviour and the process parameter enhancements, mainly in the terms of injection moulding process, are important to the future development of WPCs. The material’s flowability is the main issue in the injection moulding process. A limitation of the flow will limit the composite product design process in terms of the product thickness and length of flow. A better understanding of the processing parameters based on the PP/OPTP’s viscosity behaviour will help overcome the design limitations and enable great potential usage for WPCs.

## Figures and Tables

**Table 1:** Properties the OPT chemi-themomechanical pulps used in this study.

Pulp Yield (%)	Fibre Geometry ^1^			Chemical Composition		
	Fibre Length (mm)	Fibre Width (mm)	Fibre Aspect Ratio	Lignin (%)	Holocellulose (%)	α-Cellulose (%)
58%	1.54	0.037	41.6	20.45	73.52	43.43

Note: ^1^ Mean values based on measurements of 50 fibres.

**Table 2:** The main characteristics of the polypropylene (Homopolymer G452) used in this study.

Typical Resin Properties	Value	Unit
Melt Flow Rate (230 °C)	45	g/10 min
Density	0.9	g/cm^3^
Tensile Strength at Yield	380	kg/cm^2^
Elongation at Yield	10	%
Flexural Modulus	17,500	kg/cm^2^
Heat Deflection Temperature at 4.64 kg/cm^2^	105	°C

**Table 3:** Formulation used in producing the PP/OPTP composite.

Sample ID	Material Loading (%)			Type of Coupling Agent
	OPT Pulp	Plastic (PP)	Coupling Agent	
65/33/2EX	65	33	2	Exxelor PO 1020
65/32/3EX	65	32	3	Exxelor PO 1020
65/31/4EX	65	31	4	Exxelor PO 1020
65/33/2CA	65	33	2	CA 10512
65/32/3CA	65	32	3	CA 10512
65/31/4CA	65	31	4	CA 10512
65/33/2EP	65	33	2	Epolene E-43
65/32/3EP	65	32	3	Epolene E-43
65/31/4EP	65	31	4	Epolene E-43
70/28/2EX	70	28	2	Exxelor PO 1020
70/27/3EX	70	27	3	Exxelor PO 1020
70/26/4EX	70	26	4	Exxelor PO 1020
70/28/2CA	70	28	2	CA 10512
70/27/3CA	70	27	3	CA 10512
70/26/4CA	70	26	4	CA 10512
70/28/2EP	70	28	2	Epolene E-43
70/27/3EP	70	27	3	Epolene E-43
70/26/4EP	70	26	4	Epolene E-43
75/23/2EX	75	23	2	Exxelor PO 1020
75/22/3EX	75	22	3	Exxelor PO 1020
75/21/4EX	75	21	4	Exxelor PO 1020
75/23/2CA	75	23	2	CA 10512
75/22/3CA	75	22	3	CA 10512
75/21/4CA	75	21	4	CA 10512
75/23/2EP	75	23	2	Epolene E-43
75/22/3EP	75	22	3	Epolene E-43
75/21/4EP	75	21	4	Epolene E-43

**Table 4:** Injection moulding process conditions for the optimisation study.

Processing Section	Setting	Condition
Extruder	Barrel Temperature	180 °C
	Extruder output speed	5 rpm
Press Cylinder	Temperature	180 °C
	Heating time	10 min
Mould	Temperature *	25, 50 or 80 °C
Hydraulic Press Machine	Pressure *	40 or 50 tonnes
	Pressing Time	10 s
	Holding Time	4–5 min

Note: * The injection moulding process parameters considered in this study were the mould temperature and pressing pressure.

**Table 5:** Moisture content and density values of PP/OPTP composites with different material loading formulations.

Pulp Loading (%)	Type of Coupling Agent	Coupling Agent (%)	Moisture Content (%)	Density (g/cm^3^)
65	Exxelor PO 1020	2	0.70 (0.20)	1.18 (0.01)
65	Exxelor PO 1020	3	0.47 (0.09)	1.13 (0.01)
65	Exxelor PO 1020	4	0.72 (0.15)	1.11 (0.01)
65	CA 10512	2	0.59 (0.06)	1.13 (0.02)
65	CA 10512	3	0.54 (0.06)	1.18 (0.04)
65	CA 10512	4	0.53 (0.04)	1.15 (0.03)
65	Epolene E-43	2	0.67 (0.08)	1.15 (0.04)
65	Epolene E-43	3	0.58 (0.06)	1.13 (0.04)
65	Epolene E-43	4	0.49 (0.05)	1.18 (0.02)
70	Exxelor PO 1020	2	0.79 (0.15)	1.21 (0.01)
70	Exxelor PO 1020	3	0.80 (0.32)	1.13 (0.01)
70	Exxelor PO 1020	4	0.86 (0.40)	1.13 (0.03)
70	CA 10512	2	0.77 (0.06)	1.17 (0.03)
70	CA 10512	3	0.79 (0.05)	1.17 (0.03)
70	CA 10512	4	0.65 (0.05)	1.12 (0.04)
70	Epolene E-43	2	0.81 (0.06)	1.18 (0.02)
70	Epolene E-43	3	0.74 (0.04)	1.14 (0.02)
70	Epolene E-43	4	0.72 (0.05)	1.15 (0.03)
75	Exxelor PO 1020	2	0.86 (0.17)	1.22 (0.01)
75	Exxelor PO 1020	3	0.93 (0.13)	1.14 (0.03)
75	Exxelor PO 1020	4	0.92 (0.25)	1.14 (0.04)
75	CA 10512	2	0.94 (0.12)	1.19 (0.04)
75	CA 10512	3	0.95 (0.13)	1.18 (0.04)
75	CA 10512	4	0.97 (0.17)	1.14 (0.06)
75	Epolene E-43	2	0.95 (0.08)	1.16 (0.03)
75	Epolene E-43	3	0.91 (0.10)	1.18 (0.04)
75	Epolene E-43	4	1.02 (0.14)	1.14 (0.06)
*p*-value			ns	ns

Note: The values in parentheses are the standard deviations of the mean values. Note: ns, not significant at *p* > 0.05.

**Table 6:** Physical and mechanical properties of PP/OPTP composites from different material loading formulations.

Pulp Loading (%)	Type of Coupling Agent	Coupling Agent (%)	Water Adsorption (%)	MOR (MPa)	MOE (MPa)
65	Exxelor PO 1020	2	1.49(0.01) ^cdef^	23.7(0.29) ^efgh^	2094(4.36) ^m^
65	Exxelor PO 1020	3	0.85(0.02) ^a^	24.6(0.21) ^efg^	2278(1.73) ^ijklm^
65	Exxelor PO 1020	4	1.05(0.01) ^ab^	26.2(0.20) ^cde^	2560(2.65) ^efgh^
65	CA 10512	2	1.54(0.05) ^cdefg^	28.4(0.14) ^cd^	2255(7.55) ^jklm^
65	CA 10512	3	1.43(0.03) ^cdef^	28.8(0.40) ^cd^	2310(5.57) ^hijklm^
65	CA 10512	4	1. 32(0.03) ^bcde^	29.2(0.12) ^c^	2375(3.05) ^ghijkl^
65	Epolene E-43	2	1.64(0.12) ^fgh^	22.5(0.16) ^fgh^	2560(2.08) ^efgh^
65	Epolene E-43	3	1.76(0.02) ^fgh^	23.4(0.20) ^efgh^	2585(1.10) ^efg^
65	Epolene E-43	4	1.26(0.05) ^bcd^	23.6(0.21) ^efgh^	2604(2.00) ^defg^
70	Exxelor PO 1020	2	2.26(0.02) ^jk^	20.4(0.19) ^h^	2131(4.00) ^lm^
70	Exxelor PO 1020	3	1.98(0.08) ^hij^	25.6 (0.37) ^def^	2568(3.51) ^efgh^
70	Exxelor PO 1020	4	1.18(0.04) ^abc^	63.3(0.17) ^a^	4275(2.65) ^a^
70	CA 10512	2	2.32(0.07) ^jkl^	25.4(0.06) ^def^	2430(2.52) ^fghijk^
70	CA 10512	3	1.87(0.11) ^ghi^	25.8(0.03) ^cdef^	2510(3.22) ^efghij^
70	CA 10512	4	1.45(0.01) ^cdef^	25.9(0.02) ^cdef^	2505(4.58) ^efghij^
70	Epolene E-43	2	2.15(0.03) ^ijk^	21.5(0.10) ^gh^	2860(4.16) ^bcd^
70	Epolene E-43	3	1.6(0.02) ^defg^	21.4(0.27) ^gh^	2966(4.04) ^b^
70	Epolene E-43	4	1.54(0.03) ^cdefg^	21.3(0.14) ^gh^	2898(2.65) ^bc^
75	Exxelor PO 1020	2	2.78(0.03) ^no^	21.3(0.13) ^gh^	2224(7.00) ^klm^
75	Exxelor PO 1020	3	2.96(0.02) ^p^	22.7(0.08) ^efgh^	2520(3.00) ^efghi^
75	Exxelor PO 1020	4	2.25(0.06) ^jk^	42.2(0.35) ^b^	4240(8.02) ^a^
75	CA 10512	2	2.83(0.06) ^p^	22.5(0.28) ^fgh^	2512(3.06) ^efghij^
75	CA 10512	3	2.45(0.02) ^lmn^	23.1(0.17) ^efgh^	2670(2.52) ^cdef^
75	CA 10512	4	2.21(0.02) ^ijk^	24.5(0.47) ^efg^	2695(4.58) ^cde^
75	Epolene E-43	2	2.66(0.04) ^mno^	20.2(0.32) ^h^	2756(4.36) ^bcde^
75	Epolene E-43	3	2.38(0.01) ^lm^	20.3(0.10) ^h^	2876(1.53) ^bc^
75	Epolene E-43	4	2.23(0.01) ^ijk^	21.2(0.29) ^gh^	2925(3.00) ^bc^
*p*-value			***	***	***

Note: Means followed by the same letters in the same column were not significantly different at *p* ≤ 0.05 according to Tukey’s test; *** significant at *p* ≤ 0.01.

**Table 7:** Moisture content and density values of PP/OPTP composites with different injection moulding parameters.

Pulp Loading (%)	Mould Temp. (°C)	Pressing Pressure (ton)	Moisture Content (%)	Density (g/cm^3^)
65	RT	40	0.72 (0.16)	1.11 (0.01)
65	RT	50	0.72 (0.12)	1.15 (0.02)
65	50	40	0.68 (0.07)	1.15 (0.04)
65	50	50	0.69 (0.08)	1.18 (0.05)
65	80	40	0.66 (0.03)	1.17 (0.03)
65	80	50	0.66 (0.06)	1.21 (0.07)
70	RT	40	0.86 (0.41)	1.13 (0.04)
70	RT	50	0.84 (0.05)	1.14 (0.03)
70	50	40	0.81 (0.05)	1.16 (0.02)
70	50	50	0.80 (0.32)	1.18 (0.04)
70	80	40	0.75 (0.05)	1.18 (0.04)
70	80	50	0.72 (0.03)	1.22 (0.09)
75	RT	40	0.92 (0.25)	1.14 (0.03)
75	RT	50	0.91 (0.11)	1.16 (0.03)
75	50	40	0.85 (0.08)	1.16 (0.03)
75	50	50	0.84 (0.06)	1.20 (0.05)
75	80	40	0.82 (0.06)	1.19 (0.05)
75	80	50	0.76 (0.05)	1.22 (0.03)
*p*-value			ns	ns

Note: The values in parentheses are the standard deviation of the mean values; RT: room temperature; ns: not significant at *p* > 0.05.

**Table 8:** The physical and mechanical properties of PP/OPTP composites with different injection moulding parameters.

Pulp Loading (%)	Mould Temp. (°C)	Pressing Pressure (ton)	Water Adsorption (%)	MOR (MPa)	MOE (MPa)
65	RT	40	1.05(0.02) ^ab^	26.2(0.96)	2560(4.36)
65	RT	50	1.03(0.02) ^ab^	26.8(0.05)	2575(9.64)
65	50	40	0.96(0.01) ^a^	27.5(1.04)	2745(3.60)
65	50	50	0.97(0.01) ^a^	27.8(0.58)	2816(3.46)
65	80	40	0.90(0.03) ^a^	27.6(1.12)	2786(6.08)
65	80	50	0.91(0.03) ^a^	28.2(0.35)	2913(2.08)
70	RT	40	1.18(0.12) ^bc^	63.3(0.14)	4272(3.06)
70	RT	50	1.19(0.02) ^bc^	63.5(0.34)	4238(5.51)
70	50	40	1.06(0.02) ^ab^	64.8(0.90)	4390(4.36)
70	50	50	1.01(0.01) ^ab^	65.2(0.11)	4552(14.17)
70	80	40	0.99(0.01) ^ab^	66.5(0.15)	4718(7.55)
70	80	50	0.94(0.04) ^a^	67.6(0.88)	4898(13.05)
75	RT	40	2.25(0.07) ^f^	42.2(1.00)	4240(3.00)
75	RT	50	1.94(0.11) ^e^	42.8(0.15)	4275(4.58)
75	50	40	1.55(0.03) ^d^	45.7(0.13)	4415(4.00)
75	50	50	1.42(0.02) ^d^	46.8(0.61)	4521(2.65)
75	80	40	1.50(0.06) ^d^	47.8(1.07)	4585(3.46)
75	80	50	1.38(0.02) ^cd^	49.6(0.22)	4725(10.69)
*p*-value			***	ns	ns

Note: Means followed by the same letters in the same column were not significantly different at *p* ≤ 0.05 according to Tukey’s test. Note: ns: not significant at *p* > 0.05; *** significant at *p* ≤ 0.01.

**Table 9:** Summary of the least significant difference (LSD) test results for the different pulp loadings based on the flexural properties of the WPCs.

Pulp Loading (%)	Flexural Properties	
	MOR (MPa)	MOE (MPa)
65	27.4 ^c^	2732 ^b^
70	65.2 ^a^	4512 ^a^
75	45.8 ^b^	4460 ^a^
*p*-value	***	***

Note: Means followed by the same letters in the same column were not significantly different at *p* ≤ 0.05 according to Tukey’s test. Note: *** significant at *p* ≤ 0.01.

**Table 10:** Summary of the least significant difference (LSD) test results for the mould temperatures based on flexural properties of the PP/OPT composites.

Mould Temperature (°C)	Flexural Properties	
	MOR (MPa)	MOE (MPa)
RT	44.2 ^b^	3694 ^c^
50	46.3 ^a^	3907 ^b^
80	47.9 ^a^	4104 ^a^
*p*-value	***	***

Note: Means followed by the same letters in the same column were not significantly different at *p* ≤ 0.05 according to Tukey’s test. Note: *** significant at *p* ≤ 0.01.

**Table 11:** Comparison of the current study and other published studies based on the properties of WPCs reinforced with pulp.

Types of Filler	Pulp Loading	Type and Loading % of Polymer	Type and Loading % of Coupling Agent	Forming Method	Density (g/cm^3^)	Water Absorption (%)	MOR (MPa)	MOE (MPa)	References/ Trade Name
CTMP (*Elaeis guineensis*)	70%	PP (26%)	Maleated PP (4%)	Injection Moulding	1.22	0.9	68	4898	Current study
TMP (*Pinus sylvestris*)	50%	HDPE (47%)	Maleated HDPE (3%)	Injection Moulding	1.10	1.4	63	3750	[12]
CTMP (White birch)	40%	HDPE (60%)	Not added	Injection Moulding	1.08	3.9	44	3390	[14]
CTMP (White birch)	39%	HDPE (59%)	Maleated PE (2%)	Extrusion	1.08	6.5	40	2830	[13]
CTMP (White birch)	38%	HDPE (57%)	Maleated PE (2%)	Injection Moulding	1.02	5.3	51	3390	[13]
CTMP (*Pinus resinosa*)	30%	HDPE (70%)	Not added	Extrusion	NA	NA	≈33	≈1900	[42]
TMP (*Pinus sylvestris*)	70%	PP (26%)	Maleated PP (3%)	Extrusion	NA	NA	38	4927	[41]
TMP (*Robinia pseudoacacia*)	70%	PP (26%)	Maleated PP (3%)	Extrusion	NA	NA	39	4268	[41]
TMP (*Fagus sylvatica*)	60%	HDPE (37%)	Maleated PP (2%)	Injection Moulding	NA	NA	53	4178	[43]
Recycled hardwood	60%	Recycled plastic (27%)	xx	Extrusion	1.30	0.5	34	4637	Dream Living^TM^ Dura Deck Eco
xx	xx	xx	xx	Extrusion	NA	NA	32	5515	Fiberon Wildwood^TM^
xx	xx	xx	xx	Extrusion	NA	0.2	25	3495	NewTechWood^TM^
xx	xx	xx	xx	Extrusion	0.98	<2.5	17	1850	UPM ProFi Lifecycle^TM^

Note: xx: not specified; NA: not available; ^TM^: Trademark.

## Data Availability

All relevant data are within the manuscript.
